# Understanding Food Loss and Waste—Why Are We Losing and Wasting Food?

**DOI:** 10.3390/foods8080297

**Published:** 2019-07-29

**Authors:** Rovshen Ishangulyyev, Sanghyo Kim, Sang Hyeon Lee

**Affiliations:** 1 Department of Information, Turkmen Agricultural Institute, Dashoguz 746300, Turkmenistan; 2 Division of Food and Marketing Research, Korea Rural Economic Institute, Naju 58217, Korea; 3 Department of Agricultural & Resource Economics, Kangwon National University, Chuncheon 24341, Korea

**Keywords:** food loss, food waste, waste management, waste prevention, food security

## Abstract

The Food and Agricultural Organization (FAO) reported that approximately one-third of all produced foods (1.3 billion tons of edible food) for human consumption is lost and wasted every year across the entire supply chain. Significant impacts of food loss and waste (FLW) have increased interest in establishing prevention programs around the world. This paper aims to provide an overview of FLW occurrence and prevention. Economic, political, cultural, and socio-demographic drivers of FLW are described, highlighting the global variation. This approach might be particularly helpful for scientists, governors, and policy makers to identify the global variation and to focus on future implications. The main focus here was to identify the cause of the FLW occurrence throughout the food supply chain. We have created a framework for FLW occurrence at each stage of the food supply chain. Several feasible solutions are provided based on the framework.

## 1. Introduction

Food loss and waste (FLW) is recognized as a serious threat to food security, the economy, and the environment [1]. Approximately one-third of all food produced for human consumption (1.3 billion tons of edible food) is lost and wasted across the entire supply chain every year [2]. The monetary value of this amount of FLW is estimated at about USD $936 billion, regardless of the social and environmental costs of the wastage that are paid by society as a whole [3]. The amount of FLW is sufficient to alleviate one-eighth of the world’s population from undernourishment [2] and address the global challenge to satisfy the increased food demand, which could reach about 150–170% of current demand by 2050 [4]. 

The amount of FLW varies between countries, being influenced by level of income, urbanization, and economic growth [5]. In less-developed countries, FLW occurs mainly in the post-harvest and processing stage [2], which accounts for approximately 44% of global FLW [6]. This is caused by poor practices, technical and technological limitations, labor and financial restrictions, and lack of proper infrastructure for transportation and storage [2]. The developed countries, including European, North American, and Oceanian countries, and the industrialized nations of Japan, South Korea, and China produce 56% of the world FLW [6]. Of this, 40% of FLW in developed countries occurs in the consumption stage [2], which is driven mostly by consumer behavior, values, and attitudes [7]. A large portion of the food waste occurs after preparation, cooking, or serving, as well as from not consuming before the expiration date as a result of over-shopping, which might be associated with poor planning and bulk purchasing [7,8]. The amount of Food Waste (FW) in industrialized countries, at approximately 222 million tons, is almost equal to the total net production in Sub-Saharan African (SSA) counties (230 million tons) [2]. 

FLW is a critical concern in terms of nutritional insecurity, as it decreases the availability of food for human consumption. FLW also has serious environmental, economic, poverty, and natural resource impacts [6]. When FW is thrown into landfills, a substantial portion of FW is converted into greenhouse gas (GHG) and methane, which has a global warming potential 25 times higher than carbon dioxide [9]. FW decomposes faster than other landfilled materials, with a higher methane yield and without any contribution to biogenic sequestration in that area [10]. According to Rutten [11], FLW represents dissipated investment in the agricultural sector and generates significant inefficiencies in the input aspects, such as land, labor, water, fertilizers, and energy. Several studies also showed that FLW reduction initiatives in developed countries could decrease food prices in developing countries [11], boost efficiency in their supply chain, and conserve resources that might be used to feed the hungry [12]. Such changes could lead to improved access to nutritious foods for vulnerable households [2].

FLW has more recently become a substantial issue, as confirmed by the fact that the number of research publications has dramatically increased since the late 2000s [1]. FLW is an interdisciplinary subject that integrates studies from diverse fields ranging from agricultural and environmental studies to logistics and business [13]. Many studies have examined the main drivers of FLW at stages of the food supply chain (FSC) or as a whole, and systematic reviews of these studies have also been conducted. For example, Lipinski et al. [6] examined the global efforts and policy implications of reducing FLW. Abiad and Meho [1] conducted a systematic review of FLW research in the Arab world. They found that, in the Arab world, there is insufficient concern, initiatives, and research related with FLW or its reduction. Schneider [14] reviewed literature on FLW prevention at the global level. Their main finding was the limitations of research, such as lack of a consistent definition for FLW, absence of information for food loss (FL) in the transportation stage, and undeveloped methodologies of studies of FLW prevention. Another review was conducted by Thyberg and Tonjes [15] about the drivers of FLW and their implications for sustainable policy development. 

The purpose of this paper was to provide a broad picture of FLW generation and prevention. Our goals are: (1) to investigate the importance and status of FLW by reviewing previous studies, which will help in understanding the negative effects of FLW and why prevention activities are necessary; (2) to investigate FLW reduction policy trends, which will answer questions such as “What kinds of programs have been implemented for the reduction and prevention of FLW?”; and (3) to investigate reasons for the occurrence of FLW along the FSC. 

We searched for previous studies that addressed the FLW issue. The structure of the systematic review followed the Preferred Reporting Items for Systematic Reviews and Meta-Analyses (PRISMA) which are suggested by Moher et al. [16]. To understand the policy trends, government and international organization websites (United Nations (UN), Food and Agriculture Organization (FAO), International Fund for Agricultural Development (IFAD), European Union (EU), etc.) were reviewed. For the investigation of the FLW studies along the FSC, the databases that were used during the search process were Scopus, Science Direct, Jstor, and Google Scholar. Keyword searches included “food loss and waste”, “food loss”, “food waste”, and “food supply chain”. The Google search engine was used to find relevant documents from institutions. Some other documents were obtained by examining reference lists and citations of key articles. Articles covering the period 2001–2018 were reviewed. The number of articles initially obtained through the database search was 82,730. Based on title and abstract screening, we excluded articles that were not relevant to FLW generation and prevention. Among 264 articles left after title and abstract screening, we excluded an additional 145 articles that only covered the technical aspect of FLW generation. Therefore, 119 articles were included in this systematic review. 

## 2. Definitions and Situations of FLW

To date, no commonly agreed-upon definition of FLW exists [17], thus it has been difficult to measure FLW, to conduct associated research, and to determine the exact policy objectives. Various terms, such as food waste, food loss, post-harvest loss, spoilage, food and drink waste, bio-waste, and kitchen waste, are used interchangeably (see Table 1) [14]. These terms can be used to express totally different concepts [18]. One of the main problems occurs when such terms are translated into another language, especially from the author’s native language to English for international publication [14]. However, several institutions have announced and used their own definitions in their studies as follows. 

The FAO defined FL as decrease in weight (dry matter) or quality (nutritional value) of food that was originally produced for human consumption. Most of those losses are resulted from inefficiencies created along the FSC, such as poor logistics and infrastructure, scarcity of technology, knowledge, skills, and management capacity of supply chain participants, and lack of market access. 

FW was defined by the FAO as food appropriate for human consumption being discarded, whether after it is left to spoil or kept beyond its expiry date. This is often due to the foods that have been spoiled, but there can be some other reasons, such as oversupply, depending on the market conditions, or individual consumer eating and shopping habits [19]. 

The Food Use for Social Innovation by Optimising Waste Prevention Strategies EU (FUSIONS EU) project has defined FW as “Any food and its inedible parts, removed from the FSC to be disposed (including composted, crops ploughed in or not harvested, anaerobic digestion, bio-energy production, co-generation, incineration, disposal to sewer, landfill, or discarded to sea) or recovered” [20].

High Level Panel of Experts (HLPE) defined FL as, “A decrease, at all stages of the FSC prior to the consumer level, in mass of food that was originally intended for human consumption, regardless of the cause”, and they defined FW as “food appropriate for human consumption being discarded or left to spoil at consumer level—regardless of the cause” [21]. 

The United States Department of Agriculture (USDA) defined FLW as, “FW is a subcomponent of FL and occurs when an edible food goes unconsumed. The food which is still edible at the time of discard is considered as food waste” [22]. 

The above listed definitions are all similar in expressing the decrease in the quantity or quality of food aimed for human consumption. However, they have differences in considering the external causes and defining the relationship between FW and FL (Table 1). According to the FAO, FL occurs during the first three stages of the FSC, and FW means the wastage that occurs at the final stage of the FSC. According to this definition, FW is related to retailer and consumer behavior. For FUSIONS EU, all losses and waste refer to FW; there is no FL terminology. HLPE determines FL as a decrease during the first four stages of the FSC and FW refers to a decrease only in the final stage of the FSC. This definition, FW, is related only to consumer behavior. USDA interprets FW as a subset of FL, and FL is a decrease in food throughout the FSC. 

**Figure 1:**
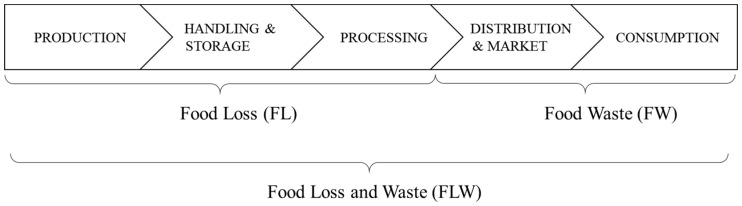
Framework of Food Loss and Waste (FLW) definitions.

The definitions used in this study (Figure 1) are similar to those of the FAO as follows. FL is reduction in edible food weight throughout the first three stages of the FSC. The drivers for loss considered in this study include infrastructure limitations, environmental factors, and quality or safety standards. FW is food that is produced or processed originally for human consumption but is not consumed by a person. FW includes foods that were edible when thrown and spoiled before disposal. Basically, FW represents discard that occurs in distribution, marketing, and consumption stages. However, in this study, external causes were not considered so that we could focus on FW or FL generation in the FSC.

In defining FLW, as well as suggesting ways to reduce FLW, avoidable FLW should be distinguished from unavoidable FLW. Unavoidable FLW is reprensented by the types of foods that cannot be in general eaten by human beings, including meat bones and the skin of watermelons. On the other hand, avoidable FLW occurs for the types of foods that could have been used or eaten at some point of the FSC but neither used nor eaten. It is clear that the policy efforts to prevent and reduce FLW, as well as future studies, should focus on avoidable FLW. For example, food policies that prevent foods that can be eaten today but can not be eaten tomorrow being lost and wasted through ways such as temporal or spatial movement of the foods or dietary education could be more effective in reducing FLW. Even though it is not impossible to research and develop a technology or machine transforming the skins of watermelon, which has been known to be generally inedible, into a food that is edible, focusing on relatively unavoidable FLW could be a more ineffective way to reduce FLW. 

### 2.1. FLW Quantifications

Quantifying the level of FLW is important for the development of well-planned and effective policies and programs, which can be used to distinguish the changes in residual flows after FLW prevention and recovery policies are implemented [23]. Understanding the impact of FLW can provide people with motivation to change their attitudes and behaviors. However, the absence of an exact quantification method leads to a data problem [24]. Various methods have been used for quantifying FLW (Table 2), all of which have their own weaknesses. For example, some approaches only count the amount of food that is wasted in the municipal solid waste (MSW), such as waste from irrelevant sectors [15] (Table 2). Other methods focus on the overall amount of FLW generated from particular sectors, such as households and restaurants, or aim to link wasted quantity with behavioral action. However, measuring the FLW based on this method is challenging—consumers mostly underestimate their waste when they are surveyed. For example, in Spain, according to a survey, FW was estimated at 4% of food, while the actual amount was 18% [13]. Some FW studies focused on excluded wastes, which disappear through the system of waste management, such as food-fed animals, compost in home, and waste placed down the drain [15]. A study that examined an Australian case estimated that 20% of Australian FLW flow is due to informal food disposal [25].

Table 3 expresses the global and country-specific estimated FLW quantities and shows the diversity in the scale, scope, and quantification of these methods. Table 2 shows the differences in estimated FLW quantities by area. For instance, estimated annual FLW quantity per capita is 637 kg in Australia and 177 kg in South Africa. It is difficult to compare FLW quantities between studies or between countries because studies have applied different criteria for FLW quantification. Therefore, a consistent quantification method is required. Recent studies, such as those by Hanson et al. [26], Östergren et al. [20], and Thyberg et al. [23] were conducted to standardize and improve quantification methods; however, estimates are heterogeneous by methodology and definition. 

### 2.2. Costs and Effects of FLW

All the actors in the FSC are economically affected by FLW. Since economic factors have been reported as the most effective motivation for FLW, the behavior of the actors can be changed if they realize the effect of FLW prevention [38]. Table 4 summarizes the economic costs of FLW. In Germany, the economic loss was calculated to be about USD $331 per capita, accounting for about 12% of expenditure on non-alcoholic beverages and food per consumer [46]. Buzby and Hyman [12] found that in 2008, the per capita amount of FW was 124 kg, which is monetarized to USD $390 at the retail and consumption stages in US. Average U.S. families spend USD $1410 each year for foods that are never consumed [47]. These estimations and figures show that reduction of FLW is important because FLW is associated with the possibility of inefficiently using scarce resources and preventing financial losses.

There is increasing awareness that important environmental burdens are related to FSC. Food production affects the environment by harming plants, animals, and ecosystems as a whole [18]. Imported and non-seasonal foods increase transportation and energy use. Processing of food requires more material input and energy. Additionally, the environment is more affected when demand increases for resource intensive foods (e.g., meat). FLW puts water, soil, and air at risk because food production and distribution requires large amounts of water, land, and energy [48]. The largest usage of water and input resources is food production [49]. The food production and supply system directly influences land quality, including soil erosion, desertification, deforestation, and nutrient depletion [50]. The waste of resources caused by global FLW has been estimated to account for 24% of the total usage of freshwater resources and 23% of the global fertilizer use [27]. The reduction in FLW means that it can save resources used for production, processing and transportation, which provides benefits to the environment. 

## 3. FLW in the Supply Chain

FLW occurs as a natural result of various faults throughout the FSC [54]. Throughout the FSC, millions of tons of foods are produced, processed, and transported to feed the world’s population. However, 815 million people, mostly living in developing countries, are undernourished and hungry (12.9% of total population) [55]. In the United States alone, 15.8 million households were considered as food insecure [56]. Reducing FLW by only 15% would feed all insecure U.S. households. If FLW is reduced by 50% of FLW, an additional one billion people could be fed [57]. Given the increasing demand for food, there is a serious concern related to adequate and sustainable global food supply. If the same level of FLW continues, then the soil, oceans, forests, bio-diversity, and fresh water might be in serious danger [55]. 

Efforts to reduce FLW have to start by first distinguishing where it occurs. The FAO [19] provides information about the moments when food products in the supply chain are converted to FLW: (1) crops are ripe in the plantation, field, or orchard; (2) animals are on the farm (field, pen, sty, shed, and coop) ready for slaughter; (3) milk that is drawn from the udder; (4) aquaculture fish are growing in the pond; and (5) when wild fish are caught. The supply chain ends at the point when food products are consumed, discarded, or removed for human consumption from the chain. Consequently, food that was initially produced for human consumption but removed from the chain is considered FLW, even though it could be later used as bioenergy or animal feed [19]. 

**Figure 2:**
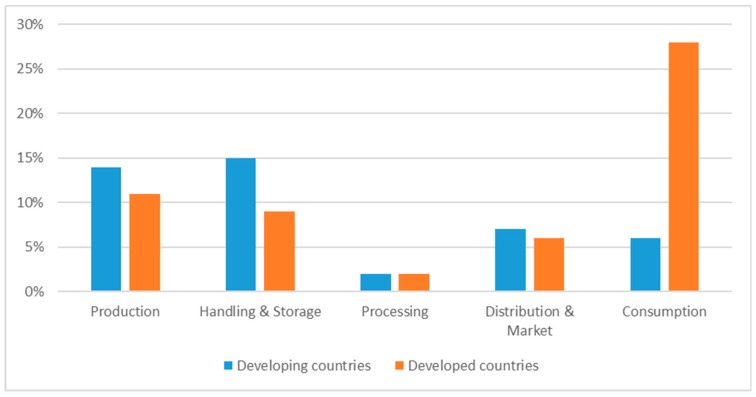
Portion of FLW in the stages of the food supply chain (FSC). Source: Lipinski et al. [6].

The UN FAO and World Resources Institute (WRI) on global FLW highlight the significant differences in per capita FLW between economies [2]. About 56% of the total FLW occurs in developed countries, while the other 44% occurs in developing countries (Table 5). However, the generated FLW varies in each stage (Figure 2). These differences are observable between developed and developing countries. Developing countries have relatively high FL, while developed countries have a higher portion of FW. 

In developing regions, 29% of FLW occurs during the first two stages (production, and handling and storage) [2]. However, in developed countries, FL occurs less in the production stage compared to developing regions, but FL in developed countries occurs due to the excessive loss of the embedded resources [58]. In both regions, the most resource-intensive stage is the production stage. That is why food sustainability models (Environmental Protection Agency’s Food Recovery Hierarchy) emphasize the reduction of food surplus generated during the production stage. FW at the consumption stage in developing regions is significantly lower due to limited household income and poverty. Households in developing countries purchase less and smaller amount of food, and they have a tendency to buy food on a daily basis [2]. For example, in the EU and North America (NA), consumer FLW per capita ranges between 95 and 115 kg, while total FLW per capita in developing regions (Sub-Saharan Africa (SSA) and South East Asia (SEA)) is between 6 and 11 kg [55].

Occurrence of FW during the last stage of FSC is generally considered more harmful. As food travels along the FSC, resources are required to move the food from stage to stage. Thus, FLW that occurs at the last stage has required more resources. In developed countries, a large portion of FLW occurs at the last stage of the FSC. Targeting FW interventions at the consumption stage may result in a significant reduction in wastage and decrease the environmental impacts of FW [2].

### 3.1. FL During Production Stage

FL occurs when appropriate access to harvesting equipment, pesticides and fertilizers, farmer training courses, extended service, and research, financial, and meteorological institutions is difficult. Harvesting method (mechanical or manual) and timing are two important factors causing the FL in this stage. Because of low mechanization rate and insufficient labor force, food loss occurs due to delayed harvesting in the harvest season [59,60]. Sometimes harvesting time is delayed due to economic reasons. Producers prefer to leave the crop without harvesting if, at that moment, demand is low and returns to harvest cannot cover the cost of harvest and transportation [3]. In addition, poor harvesting methods and equipment with poor performance can lead to food loss [61,62]. Farmers often overproduce in order to protect against pest attacks, weather, and market uncertainties, and to guarantee the contractual obligation with the buyers. Oversupply decreases the market price and leads to more crops left unharvested [2,43,63,64]. Some products are not harvested or thrown out directly after harvest because they failed to meet quality standards, such as shape, size, color, and weight, required by processors or target markets [34,64,65,66]. Poor nutrient and water management contributes to lower quality of production, resulting in high FL during the grading process. 

In the case of vegetables, fruits, and meats, product quality at the production stage heavily depends on agronomic practices, diseases, and education [20]. Poor practices can result in high FL. Pre-harvest pest infestation is one of the major factors causing post-harvest FL for fruits and vegetables, as some of the infestations begin to appear after harvesting [67]. In meat production, FL occurs due to death during breeding, which can be due to poor practice and lack of knowledge [2]. One of the main causes of FL during the production stage is choosing the right variety that is adapted to a given location and meeting market requirements [68]. Choosing the wrong variety leads to the production of inferior quality food, which results in larger losses in farmer income. For cereals, such as wheat, maize, sorghum, and rice, selecting the wrong varieties that are prone to logging in locations where wind is prevalent leads to high losses. 

Consequently, the main drivers of FL in this stage are listed in Table 6. 

### 3.2. FL during Handling and Storage Stage

In this stage, occurrence of FL varies depending on the type of food product [2,69,70]. Products such as vegetables experience losses due to degradation and spillage in loading and unloading, transportation (from farm to distribution), and storage. For meat products, losses include death during handling to slaughter and condemnation in the slaughterhouse. For fish products, losses refer to degradation and spillage during icing, storage, package, and transportation after landing. Milk is also similar to fish, as losses for milk include degradation and spillage during transportation from farm to distribution. FL during handling and storage stages accounts for the largest portion of the total FLW. Due to the poor transportation infrastructure and improper transportation vehicles, fresh products such as fish, meat, vegetables, and fruits can easily perish in hot weather due to absence of infrastructure for transportation and improper vehicles [65]. The FL level in transportation can be relatively low with good road infrastructure, facilities in the fields, and proper loading and unloading facilities [59]. Therefore, better transportation infrastructure and loading facilities could potentially reduce FL. Timely transportation from warehouse to retail through accurate forecasting of demand is also important for reducing food loss [71,72]. If accurate timing is not achieved, the food must be stored on the retail shelf for too long, which leads to food waste by reducing the quality of the food or expiration of the consumption period.

Proper warehouses (storage facilities) help manage the time constraint, extending marketing and consumption time so that FL can be reduced [65,73]. With the absence of storage infrastructure and inaccessibility to or non-existence of cold storage facilities, highly perishable products are often discarded generating FL. Good storage conditions, which can properly control light, moisture, oxygen level, sanitation, and temperature, help reduce FL of perishable products [74]. Foods such as grain can be better stored if drying facilities are optimized [75,76]. If the storage facility is not suitable, food loss due to pests, disease, and spillage also occurs [66,77].

The main drivers of FL in the handling and storage stage are listed in Table 7.

### 3.3. Processing and Packaging Stage FL

There are some unavoidable losses that occur in the processing stage for some products, such as meat, milk, and fish [2,43,66]. For example, losses of meat occur during additional industrial processing (e.g., sausage production) and trimming spillage during slaughtering. For milk, spillage occurs during pasteurization (industrial milk treatment) and losses occur during milk processing for yogurt and cheese. For fish, losses during industrial processing and packaging (canning and smoking) can occur. However, occurrence of FL at the processing and packaging stages is mostly due to technical inefficiencies and malfunctions [21,43]. Errors in processing lead to defects in the final product, such as incorrect shape, size, weight, or packaging damage. Sometimes these kinds of defects do not seriously affect the safety and quality of the final product, although they will be discarded in accordance with established safety and quality standards [21,66]. 

Insufficient processing line capacity and inefficient processing methods can also lead to FL [21,43,78]. Failure to accurately predict demand can result in food loss if too much raw material is purchased and stored for food processing [79]. Frequent changes in the food produced in processing facilities are also the cause of food loss [43]. The contamination in a processing line that occurs due to improperly cleaned processing units not sanitized from previous processes is also one of main causes of FL occurrence, especially for animal products [43,66]. Proper process management to guarantee food quality and safety based on published standards can be a key factor in reducing FL [77]. Proper packaging also can play a significant role in extending the shelf life of food products and reducing FL [2,78]. At this stage, considerable FL is produced due to legislation restrictions on the appearance of fruits and vegetables [80]. FL also occurs during cleaning, inspection, processing, and packaging processes, and in conforming to food safety standards [74,81]. Overproduction of processed food, especially refrigerated foods with short shelf life, is one of the major causes of food waste [63,82]. 

The main drivers of FL in the processing and packaging stage are listed below in Table 8.

### 3.4. FW on Distribution and Marketing Stage

This stage includes the activity of transporting food from one place (farm, factory, storage, etc.) to another. Additionally, this stage includes the set of market activities (retail or wholesale) that allow consumers access to food. 

To avoid FW in distribution activities, it is important to use appropriate conveyance conditions, e.g., temperature-controlled aircrafts and ships, which move vegetables and fruits between continents. For products such as milk, trucks collect the milk, connecting farms with pasteurization plants. To avoid contamination, the truck’s carriage must be kept clean and hygienic [74].

In most developing countries, food loss is caused by transport through poorly maintained roads. For example, fruit is often wasted because of bruises and bumps due to road conditions. In rainy seasons, transportation of food using rural roads becomes demanding due to road blockages or landslides. However, during dry seasons, the likelihood of contamination increases due to dust [83]. When moving duration and distance are longer than the ripening process, expiry dates are shortened. Therefore, the likelihood of commercialization declines and buyers refuse some part of the delivered food. Also, in traditional markets, sellers sprinkle unclean water on vegetables and fruits to decrease the shriveling and wilting in hot weather under sunlight. This kind of technique, which aims to slow deterioration, could produce unsafe foods that are avoided by buyers and end up as landfill [65]. These phenomena happen in developing countries due to transport congestion, vehicle failures, bad weather, and lack of capital and facilities [74]. 

In developed countries, there is a commonly self-imposed rule between food businesses—called the “rule of one-third”. According to this rule, food must be delivered to suppliers at one-third of their shelf time with the main intention of providing consumers a broad choice of fresh products that are relatively far from their expiration date. However, if products are not delivered according to this rule, then many retailers refuse to buy them and return the orders, which results in the FW of safe foods [58]. Consequently, edible products are sorted out due to quality, expired before being purchased, or being damaged or spilled in the market [6,43,64,66]. In addition to the distribution stage, a similar situation occurs in the marketing stage. The owners of stores seek to manage various products displayed in large quantities and are regularly refilled to supply the shelves for consumer satisfaction. When retailers mix the same product with different expiry dates, sooner expiry dates are refused by the consumers because everyone prefers fresher products [65]. Retailers sell fresh-cut vegetables and fruits and ready-made convenience foods to meet consumer demand. However, these kinds of foods mostly have a one-day shelf life. So, if all the displayed foods cannot be sold, then these foods must be discarded. Increases in fresh-cut products have been motivated by the consumer demand for fresh, convenient, and healthy foods that are nutritious and safe. However, perishing of the fresh-cut products is accelerated by poor temperature and packaging management [66,84]. Even in developed countries with good packaging and temperature management conditions, the amount of fresh cut products that are landfilled remains high [21]. 

Commercial pressure in the marketing stage is also a major cause of food waste [66,85]. Promotional activities, such as “buy one and get one free”, which are conditional on increasing the purchase quantity, lead consumers to waste food by inducing them to purchase more food than necessary.

Given these research conclusions, the main drivers of FL in the distribution and marketing stage are listed in Table 9.

### 3.5. Consumption Stage FW

FW during this stage means the leftovers in a house, business place, or restaurant (cafeteria). Foods that are purchased and cooked but not consumed contribute to FW during this stage. Food waste in the consumption stage can be effectively reduced by future efforts [24]. According to Parfitt et al. [24], four main criteria affect FW during this stage: household size and composition, income, culture, and demographic factors. In addition to these four main factors, it has been widely confirmed in the literature that the individual attitudinal factor could also influence the FLW reduction. 

#### 3.5.1. Household Size and Composition

Household (family) size and composition play a significant role in FW generation. Households with fewer residents may discard more because the foods prepared or purchased are commonly larger than the requirements of a smaller-sized household [21]. Families with children are more likely to waste food than those without [39,86]. For instance, larger families generate less FW per capita than smaller families, particularly single-person households [86]. Koivupuro et al. [34] found people that live alone generates more FW per capita than other households. Jörissen et al. [87] also reported that single-person households generate the most FW per capita. 

In the house, FW can be generated more when enough or inadequate food is prepared [34]. In some cases, people lack food preparation skills or the ability to reuse leftovers. Approximately 40% of household FW in the United Kingdom is due to the preparation of too much food [51]. Over-provisioning could be both unintentional and intentional, as it is hard to decide how much to cook [88]. 

#### 3.5.2. Household Income

As household income increases, diets transition toward the consumption of more fruit and vegetables, diary, fish, meat, and poultry [24]. Worldwide, consumption of convenience, energy, and protein-rich foods increases along with the westernization of the Asian diet [89]. Food diversification can lead to more FW, and a more repetitive diet can lead to less FW because it is possible to reuse ingredients from one meal for another meal, using staple ingredients that are included in almost every meal [15,90]. 

Households with higher incomes tend to waste more, as food is relatively cheaper than other goods. Especially in developed countries, the proportion of expenditure on food consumption is low in the total expenditure of households, and is less sensitive to food waste during food consumption [88]. As evidence, in 2012, U.S. citizens spent 6.1% of their income on food; however, in Pakistan and Cameroon, this ratio was 47.7% and 45.9%, respectively [15]. 

#### 3.5.3. Household Demographics

Behaviors and attitudes examined in a study showed some correlation between FW and socio-demographic characteristics [88]. Examining the demographic aspects (e.g., aging population) may lead to better understanding of its relationship with FW. Although there is no clear conclusion about which socio-demographic aspects affect FW more, previous studies that examined the relationship between age and FW have shown that younger people waste more food than older people [39,51].

Hamilton et al. [91] reported that, in Australia, as age increases, FW falls sharply; young people (18–24 years old) wasted more than $30 of fresh fruit within two weeks compared with older people (70 years old and older). In the United Kingdom, people aged 65 years and over produce considerably less FW than rest of the population [51]. 

In addition, there are studies that the degree of awareness of FW is related to the actual reduction of wastage [86,88,92]. 

#### 3.5.4. Household Culture

Culture has a crucial role in dietary habits, as well as in generating FW [93]. Each culture has its own habits as to which parts of food are considered edible and which parts are thrown away, therefore, FW depends on cultural attitudes and habits [94]. For example, the United States and Australia have weak food traditions, which imply that there are fewer fundamental rituals and rules about what, when, and how to eat, and there are weak links between production, preparation, and consumption of foods [95]. Therefore, Bloom [96] argued that the United States has an unhealthy diet, and the U.S. food culture places little value on food, leading to FW. However, French food culture is different. In France, food attitudes emphasize quality rather than quantity [93], so FW is relatively lower compared to the United States. Countries that have a deep food culture tend to be more resistant to diversity, due to the strong connection between production, preparation, and consumption. Cultures that have strong connections and place higher value on food produce less FW.

Events, such as wedding, parties, and religious ceremonies, also produce FW. For instance, during Ramadan (fasting ritual) in some Arabic countries, a significant portion of prepared meals is wasted. In Saudi Arabia, 30–50% of prepared foods are wasted. Similarly, 50% in United Arab Emirates and 25% in Qatar are wasted during this time [1]. The increase in FW during Ramadan is attributed to the arrangement of extravagant meals for which the food prepared exceeds the needs of the guests and families, with leftovers becoming FW [1]. 

#### 3.5.5. Individual Attitude

The individual or household variation in the FLW can be determined by the individual’s knowledge, perceptions, and attitudes about FLW. Even if FLW is a major environmental issue that has attracted worldwide attention, it may not be a critical issue for a particular country or for a particular individual in a particular country. That is, an individual’s knowledge or attitude about the severity of the FLW problem can have a significant impact on the actual reduction, as well as the reduction intent of the FLW. The impact of attitudes and behaviors of individuals on FLW prevention could be limited, as attitudes are not entirely consistent with the actual behaviors (i.e., the attitude–behavior gap, [97]). However, there are many studies that have found evidence on the positive relationship between attitude and actual behavior on FLW reduction.

The intent to reduce or actual reduction of FLW is influenced by individual concerns about FLW. In other words, consumers who understand and concern about the severity of the FLW problem have lower FLW and the FLW reduction intention is also known to be larger [98,99,100].

Stefan et al. [101] argues that FW is influenced by consumer planning and shopping routines, and that such consumer planning and shopping routines are determined by consumer moral attitudes and perceptions. Abeliotis et al. [102] also showed that Greek consumers are very careful in the fresh food shopping stage because they show a positive attitude toward the FW prevention. This can also be explained by Marangon et al. [103]. Marangon et al. [103] confirmed that whether consumers think FW is an important issue or not has a statistically significant effect on the actual FW amount. 

In light of these findings, national campaigns and education that help appropriately shape the individual’s attitude toward reducing FLW are of great importance. In addition to global campaigns, such as UN and FAO’s “Global Initiative on Food Loss and Waste Reduction”, more country-level campaigns need to be pursued.

#### 3.5.6. Cooking Process and Method, Storage in Household, Over-Cooking

If households do not properly determine the point of purchase and purchase amount of raw materials depending on when and how much cooking is done in the household, food waste may occur [66]. In addition, the amount of food waste generated varies depending on which cooking method is selected [104]. If households do not store raw materials properly before cooking, this also causes food waste [66]. Excessive cooking in the household causes food waste, but the type of service provided in the food service industry, which provides an excessive amount of food such as a buffet, also causes food waste [43,64,105]. 

Given these research conclusions, the main drivers of FL in the Consumption stage are listed in Table 10.

## 4. Solutions and Conclusions

Creating effective solutions to reduce FLW lies in the recognition of linkages among the stages of the FSC. For instance, the performance of actors and costs of activities in upstream sections of the chain can determine the quality of the product further down the FSC [106]. In this integrated FSC approach, special attention needs to be directed to the effect of the technical interventions on the environment and the social context. However, the cost of the proposed solutions should be less than the cost of the foods that are lost or wasted [107]. Improving storage facilities on farms to reduce FLW should be integrated with a proper strategy to enhance market access. Mostly for developing countries, solutions should first consider the farmer perspective (i.e., farmer education, harvest techniques, and storage and cooling facilities) and then need to improve social infrastructures [108]. In developed (industrialized) countries, solutions in the production and processing stages can only create marginal improvements when stock management at the marketing stage and consumer awareness are absent [109]. It is important to improve communication among all stakeholders in the food supply chain, including public and private stakeholders, and to raise new awareness of food [110,111]. Information on food waste should be shared among all actors in the supply chain [110,111].

Based on our investigation, we conclude that the most important factors to reduce FLW are:

(1) Government investment in infrastructure and capacity building for agriculture;
(2) Appropriate policy implications to facilitate market access and efficient distribution methods; and
(3) Increasing awareness of FLW and establishing the right dietary habits and culture.

### 4.1. Institutional Efforts to Reduce FLW

Reducing FLW would contribute to addressing interconnected sustainability challenges, such as climate change, food security, and natural resource shortages [19]. Therefore, developing an appropriate strategy for reducing FLW is one of the important issues related to sustainable development [112]. International organizations, governments, and scholars have begun to pay more attention to FLW and its reduction. Table 11 summarizes the representative efforts.

The UN announced the Sustainable Development Goals (SDGs) agreed upon in September 2015, which identified FLW as a key challenge for achieving sustainable consumption. Goal 12.3 aims to “Halve per capita global food waste at the retail and consumer levels and reduce food losses along production and supply chains, including post-harvest losses, by 2030”, and Goal 12.5 aims to “Substantially reduce waste generation through prevention, reduction, recycling, and reuse by 2030” [55].

The Organization for Economic Cooperation and Development (OECD) created The Food Chain Analysis Network (FCAN) to focus on important issues related to the food chain and hold annual meetings, with titles such as Building a Sustainable Food Chain, Mobilizing the Food Chain for Heath, Food Waste along the Supply Chain, etc. Annual meetings began December 2010 and two meetings were devoted to the FLW issue: “Food waste along the supply chain” (June 2013) and “Reducing food loss and waste in the retail and processing sectors” (June 2016). In 2011, the FAO and Messe Dusseldorf started the “SAVE FOOD: Global Initiative on Food Loss and Waste Reduction” program. They collaborate with donors, several level agencies, financial institutions, and private sector partners to enhance and implement the FLW reduction program.

The Meeting of G20 Agricultural Chief Scientists (MACS) decided to place emphasis on FLW since 2015, and created an appropriate FLW web portal to provide information about research results regarding FLW, as well as the recent FLW innovations. Furthermore, the next MACS plan is to integrate the promising set of research findings, innovative technological solutions, and representative campaigns.

The African Postharvest Losses Information System (APHIS+) is a regional program particularly focusing on SSA countries. APHLIS+ integrates a network of local experts who supply data, a shared database, and a losses calculator. Working together, these generate estimates of the weight losses of cereal grains in SSA by country and by province [113].

Food Use for Social Innovation by Optimizing Waste Prevention Strategies (FUSIONS) is a four-year program that aims to support the 50% EU reduction target in food waste and 20% in the food chain resource inputs by 2020 through delivery of its key objectives [114]. FUSIONS deliverables are divided into five work packages split between project teams [20]. The main objectives of FUSIONS are: (1) to harmonize FW monitoring, (2) to examine the feasibility of social innovative measurements for optimized food use in the FSC, and (3) to create a Common FW Policy for EU.

In 2013, Asia-Pacific Economic Cooperation (APEC) introduced the project called “Strengthening Public-Private Partnerships to Reduce Food Losses in the Supply Chain”. Over a five-year period, this project aims to address post-harvest losses at all stages of the food supply chain in the APEC region by strengthening public-private partnerships. As part of the first step of the project, a workshop was held in 2013 in Chinese Taipei that identified key issues and challenges in post-harvest food losses, formulated a preliminary methodology on food crops, and deliberated upon strategies and action plans for APEC economies. Building upon these outcomes, expert consultations and seminars were held to strengthen public-private partnerships (PPP), to reduce food losses in the supply chain, and to tackle various topics. Examples of these seminars include Fruit and Vegetable in 2014, Fishery and Livestock in 2015, and Food Loss and Waste at the consumer level in 2016 [115].

Waste and Resources Action Program (WRAP) UK is a not-for-profit company that was established in 2000. WRAP is backed by U.K. government funding from the Department for the Environment, Food, and Rural Affairs, the Scottish Government, the Welsh Government, and the Northern Ireland Executive [6]. WRAP helps people recycle more and waste less, both at work and at home, which are practices that have economic and environmental benefits as well. In 2007, WRAP started the nationwide campaign “Love Food, Hate Waste”. Due to this campaign, the United Kingdom became fifth leading country in global FLW reduction. The campaign follows the 4E (Enable, Encourage, Engage, Exemplify) behavioral change model approach, which includes enabling people to change, engaging in the community, encouraging action, and exemplifying others’ success [116]. The model was successful, promoting a 15% reduction in household food waste and a 21% reduction in avoidable waste, which was observed from 2007 to 2012 [117]. The campaign was organized to produce this achievement by targeting consumer education and awareness using basic methods to reduce FLW [54].

There are three FLW recognition programs in the United States. These programs are operated by USDA and United States Environmental Protection Agency (USEPA). The programs are as follows: (1) The Food Recovery Challenge (FRC) was launched in 2011. The FRC was designed for organizations searching to track their FW reduction activities. Members can join as participants if they are producing FW, or as endorsers if they are not producing their own FW but can help others reduce their FLW (i.e., organizations looking to help educate or recruit for the FRC) with requirements to provide data or report activities to the challenge [118]. EPA provides a free climate report and technical assistance to participants. More than 800 participants joined this program and they have diverted food and prevented millions of tons of food from waste since it started [118]. (2) The U.S. Food Waste Challenge (USFWC) was created in 2013. The USFWC was designed for organizations seeking to make a public pledge or disclosure of their activities to reduce FW. Participants make a one-time pledge with their name and activities listed on the USDA website. The goals of the USFWC are: (a) to disseminate information about best practices to reduce, recover, and recycle FW; (b) stimulate the development of these practices across the entire U.S. FSC; and (c) provide a snapshot of the country’s commitment to and successes in reducing, recovering, and recycling FW. More than 1000 participants have joined this program as of October 2014 [118]. (3) The U.S. Food Loss and Waste 2030 Champions (USFLW) was launched in 2016. USFLW involves businesses and organizations that have made a public commitment to reduce FLW in their own operations in the United States by 50% by the year 2030. Businesses that are not ready to make the 50% reduction commitment but are engaged in efforts to reduce FLW in their operations can be recognized for their efforts by either joining FRC or the USFWC [118].

In 2015, France introduced a law banning supermarkets from throwing away unsold and unused food with the aim of decreasing food waste and increasing social welfare. Instead of wasting food, supermarkets were forced to either donate food or give it to charity [119]. As a result, France could become one of the leading countries in preventing food waste, earning the first rank in the 2017 Food Sustainability Index (FSI) [120].

### 4.2. Possible Strategies to Prevent FLW

There are many causes of FLW that we have mentioned in context of this research. Each of these causes must be addressed separately in order to develop a comprehensive strategy. Several studies have discussed strategies for FLW reduction and prevention. By comparing the research in this field, Table 12 summarizes the possible strategies to prevent FLW at different stages of the FSC.

### 4.3. Concluding Remarks

Since the late 2000s, the FLW issue has been one of the most important issues in the world. International organizations and countries have begun to implement policies to reduce FLW. Scholars are actively conducting research related to FLW. Generally, most research studies reviewed and discussed in the previous sections showed that FLW leads to economic, environmental, and social problems. All these aspects are interlinked with each other, as the emergence of one of these aspects could create another issue. The studies discussed in Section 2.2. have concluded that economic incentives are significantly associated with the environmental motivation for FLW prevention. For example, producing an edible agricultural product that cannot be sold creates FL, as this activity is economically inefficient and wastes scarce resources. However, the producer may also have used natural resources, machinery, and chemicals to provide this product. These inputs have negative effects on the environment. The studies reviewed in Section 2.2. suggested that economic and environmental issues could significantly impact society, as input resources could be used for other purposes to enhance the society. Regarding the status of FLW, the absence of a common and constant definition can lead to misunderstandings. After setting the definition, exact common quantification methods need to be determined, which will allow interested groups to obtain information about FLW. Eventually, making the process exact and clear will help with the development of well-planed, effective, and relevant policies and programs. Awareness about the impacts of FLW can provide motivation for people to change their attitudes and behaviors.

Understanding of the fact that the present food system is unstainable among scientists, institutions, businesses, policy makers, and citizens is gradually increasing. Therefore, developing appropriate strategies to reduce FLW is one of the most important issues related to sustainable development. This article has summarized the institutional efforts targeted at reducing FLW. Some of these efforts have resulted in significant reductions. France could become one of the leading countries in preventing FW, earning the first placed rank in the 2017 FSI. Other programs that were created by organizations or countries are also succeeding. For example, the SAVE FOOD program succeeded in significantly reducing FL in Kenya [93]. In Australia, the willingness of the government and actors along the FSC succeeded in reducing FW in the banana industry. Therefore, any effort targeting reduction can lead to better outcomes.

This study has deepened the understanding of FLW and emphasized that FLW is a complex problem involving various actors along the FSC. For various reasons, FLW is still remaining in each chain. Therefore, to reduce FLW in stages of the FSC, well-planned policies and programs should be created. This study presents some possible solution approaches to achieve significant outcomes in reduction. FLW is an issue that needs more and consistent attention, study, research, action, and awareness, particularly in a direction to prevent its generation.

## Figures and Tables

**Table 1:** Definitions of Food Loss and Waste.

Concepts	Definitions
Food Loss (by FAO)	Decrease in weight (dry matter) or quality (nutritional value) of food that was originally produced for human consumption
Food Waste (by FAO)	Food appropriate for human consumption being discarded, whether after it is left to spoil or kept beyond its expiry date
Food Waste (by FUSIONS EU)	Any food and its inedible parts, removed from the FSC to be disposed (including composted, crops ploughed in or not harvested, anaerobic digestion, bio-energy production, co-generation, incineration, disposal to sewer, landfill or discarded to sea) or recovered
Food Loss (by High Level Panel of Experts)	A decrease, at all stages of the FSC prior to the consumer level, in mass of food that was originally intended for human consumption, regardless of the cause
Food Waste (by High Level Panel of Experts)	food appropriate for human consumption being discarded or left to spoil at consumer level—regardless of the cause
Food Loss and Waste (by United States Department of Agriculture)	FW is a subcomponent of FL and occurs when an edible food goes unconsumed. The food which is still edible at the time of discard is considered as food waste

FAO: Food and Agriculture Organization; FUSIONS EU: Food Use for Social Innovation by Optimising Waste Prevention Strategies EU; EU: European Union; FSC: food supply chain; FW: Food Waste; FL: food loss.

**Table 2:** Summary of similarities and differences of definitions.

	Decrease in Quantity or Quality of Food	Considering External Causes	FW Subset of FL	Stages of FSC Include FL	Stages of FSC Include FW
FAO	+	+		First 3 stages	Last 2 stages
FUSIONS EU	+	+		None of them	All
HLPE	+			First 4 stages	Last stage
USDA	+	+	+	All	Last 2 stages

HLPE: High Level Panel of Experts; USDA: United States Department of Agriculture.

**Table 3:** Estimated quantities of FLW by area.

Area	Type	Amount	Reference
Global	FLW	614 kcal/person each day	[27]
Global	FLW	1.6 billion tons annually	[19]
Australia	FLW	4.06 million tons annually	[28]
Australia	FLW	637 kg/capita annually (2014–2015)	[29]
China	FLW	70% is FLW of total municipal solid waste (MSW)	[30]
China	FW	90 million tons (51% of MSW)	[31]
Denmark	FLW	700,000 tons annually	[32]
England	FW	16% of edible calories or 15% of edible drink and food purchases	[33]
Finland	FW	23 kg/person/year	[34]
Italy	FL	17.7 tons (3.25% of total production)	[35]
Italy	FW	40,000 tons annually	[36]
Japan	FLW	37.86 million tons in 2011	[37]
New Zealand	FW	148 kg/household/year	[38]
Nordic countries	FLW	40,000–83,000 tons annually	[39]
Singapore	FLW	788,600 tons (0.39 kg/day) in 2014	[40]
Singapore	FLW	809,800 tons in 2017	[41]
South Africa	FLW	177 kg/person annually	[42]
Switzerland	FLW	48% of total calories	[43]
United Kingdom	FW	4.2 million tons annually	[44]
United States	FW	34.69 million tons annually	[45]
United States	FW	35.5 million tons annually	[23]

**Table 4:** Summary the economic cost of FLW.

Type	Area	Value	Reference
Consumption	United Kingdom	$852.6 (€580) per household annually	[51]
Consumption	United Kingdom	$18.3 billion annually	[44]
Consumption	United States	$1410 average per household annually	[47]
Consumption	New Zealand	$873 million annually	[38]
Retail and consumption	United States	$165.6 billion annually, $390 per capita	[12]
Retail and consumption	Germany	$331 per capita	[46]
All stages	Canada	$21.1 billion annually	[52]
All stages	Global	$750 billion annually	[53]

Notes: All values converted to USD.

**Table 5:** Food Loss and Waste according to economy. Source: Lipinski et al. [6].

	FL	FW	FLW
Developing countries	30%	14%	44%
Developed countries	21%	35%	56%

**Table 6:** Possible causes of FLW at the production stage of FSC.

Stage	Possible Causes of FLW	Reference
Production Stage	- Infrastructural limitation	[2,21]
	- Over Production	[2,43,63,64]
	- Harvesting timing	[59,60]
	- Harvesting method (mechanical versus manual)	[61,62]
	- Pesticides and fertilizers	[67]
	- Economic problems	[3]
	- Quality Standards	[35,64,65,66]
	- Choice of variety	[68]

**Table 7:** Possible causes of FLW at the Handling and Storage stage of the FSC.

Stage	Possible Causes of FLW	Reference
Handling and Storage Stage	- Degradation and spillage according to product characteristics	[2,69]
	- Transportation from farm to distribution	[59,65,71,72]
	- Storage infrastructure	[65,66,73,74,75,76,77]

**Table 8:** Possible causes of FLW at the processing and packaging stage of FSC.

Stage	Possible Causes of FLW	Reference
Processing and Packaging Stage	- Unavoidable losses	[2,43,66]
	- Technical inefficiencies and malfunctions	[21,43,78,79]
	- Methods and changes in processing lines	[43]
	- Contamination in processing lines	[21,43,66]
	- Legislation restrictions	[2,66,72,80]
	- Packaging system	[2,65,78,81]
	- Overproduction	[63,82]

**Table 9:** Possible causes of FLW at the distribution and marketing stage of FSC.

Stage	Possible Causes of FLW	Reference
Distribution and Marketing Stage	- Inappropriate conveyance conditions (temperature-controlled aircrafts and ships)	[74]
	- Contamination of transportation	[74,83]
	- Transportation and market facilities	[65,74]
	- Road and distribution vehicles	[65,74]
	- Business Rule	[58]
	- Packaging management	[21,66,84]
	- Commercial conditions	[66,85]
	- Consumer Reference	[6,43,64,65,66]

**Table 10:** Possible causes of FLW at the consumption stage of FSC.

Stage	Possible Causes of FLW	Reference
Consumption Stage	- Household size and composition	[21,34,39,51,86,87,88]
	- Household income	[15,24,88,89,90]
	- Household demographics	[39,51,86,88,91,92]
	- Household culture	[1,93,94,95,96]
	- Individual attitude	[97,98,99,100,101,102,103]
	- Cooking process and method, storage in household, over cooking	[43,64,66,104,105]

**Table 11:** Summarize of leading efforts.

Organization (Country) and Initiative	Scope	Description
UN SDGs (Goal 12)	Global	Achieving Goal 12 requires a strong national framework for sustainable consumption and production that is integrated into national and sectoral plans, sustainable business practices, and consumer behavior, together with adherence to international norms for the management of hazardous chemicals and wastes.
OECD FCAN	OECD countries	FCAN seeks participation by national experts from government ministries and related institutions providing policy analysis.
FAO, Messe Dusseldorf SAVE FOOD	Global	FAO and Messe Düsseldorf are cooperating with donors, financial institutions, bi- and multi-lateral agencies, and private sector partners to develop and implement the program on FLW reduction.
Meeting of G20 Agricultural Chief Scientists (MACS)	Regional	MACS has created a FLW web portal to provide a variety of FLW-related information. The next plan for MACS is to integrate the promising set of research findings, innovative technological solutions, and benchmark campaigns.
FAO, EU, AfDB APHLIS+	Regional	APHLIS+ integrates a network of local experts who supply data, a shared database, and a calculator for losses.
APEC Strengthening PPP to Reduce FL in the Supply Chain	Regional	APEC has maintained this project since 2013. Goals of this project are to address post-harvest losses at all stages of the entire food supply chain in the APEC region by strengthening public-private partnerships.
EU FUSIONS	Regional	FUSIONS is a project working toward greater resource efficiency by significantly reducing food waste.
United Kingdom WRAP	National	WRAP helps people recycle more and waste less, at work and at home, which are practices that have economic and environmental benefits.
United States FRC	National	Launched in 2011. The FRC is designed for organizations searching to track their FLW reduction activities.
United States USFWC	National	Launched in 2013, the USFWC is designed for organizations seeking to make a public pledge or disclosure of their activities to reduce FLW.
United States USFLW	National	Launched in 2016, USFLW is businesses and organizations that have made a public commitment to reduce FLW.
France FW ban law	National	France introduced legislation for supermarkets banning the waste of unused and unsold foods. France became a leading country in preventing FW.

FRC: Food Recovery Challenge; SDGs: Sustainable Development Goals; OECD: Organization for Economic Cooperation and Development; FCAN: Food Chain Analysis Network; APHLIS+: African Postharvest Losses Information System; APEC: Asia-Pacific Economic Cooperation; PPP: public-private partnerships; WRAP: Waste and Resources Action Program; USFWC: U.S. Food Waste Challenge; USFLW: U.S. Food Loss and Waste 2030 Champions.

**Table 12:** Possible strategies to prevent the FLW at different stages of FSC.

Stage	Strategy
Production Stage	Government investments in infrastructure
	Improve harvesting techniques
	Improve market access
	Organize extension services and educate farmers
	Increase tax incentives for donating unsellable edible foods
Handling and Storage Stage	Improve transportation facilities
	Provide access to cheap handling and storage technologies
	Invest in storage facilities (warehouses, cold storage, etc.)
	Improve the ability and knowledge of workers to employ safe food handling practice
	Use of appropriate and clean containers for the products
Processing and Packaging Stage	Improve capacity of process line
	Improve packaging to keep food fresher for longer
	Standardize date labels to prevent consumer confusion
	Establish other ways to use peels and trimmings
	Improve the knowledge and ability of workers
	Facilitate sanitary and cleaning inspections
Distribution and Marketing Stage	Improve inventory systems
	Establish online marketplaces to facilitate sale (donation) of perishable products
	Change food date labeling practices and in-store promotions
	Improve institutions related to this stage
	Improve transportation vehicles
	Provide guidance on storage and preparation of food to consumers
	Improve the knowledge and ability of workers
	Improve market places (storage, covered areas)
	Interlink with research institutions to predict consumer demand changes
Consumption Stage	Facilitate increased donation of unsold foods from cafeterias and restaurants
	Implement consumer education and campaigns, both nationally and regionally
	Reduce portion sizes
	Provide education about home economics in education institutions and communities
	Involve women in food safe campaigns
	Effective use of leftovers
	Training for restaurant, cafeteria, and supermarket management to forecast customer demand and reflect demand in food purchasing to avoid bulk purchases
	Implement good storage practices
	Correctly interpret label dates
	Distribution of excess food to charitable groups
